# Protein Disulfide Isomerase-Like Protein 1-1 Controls Endosperm Development through Regulation of the Amount and Composition of Seed Proteins in Rice

**DOI:** 10.1371/journal.pone.0044493

**Published:** 2012-09-06

**Authors:** Yeon Jeong Kim, Song Yion Yeu, Bong Soo Park, Hee-Jong Koh, Jong Tae Song, Hak Soo Seo

**Affiliations:** 1 Department of Plant Science, Research Institute for Agriculture and Life Sciences, Seoul National University, Seoul, Korea; 2 School of Agricultural Biotechnology, Seoul National University, Seoul, Korea; 3 Plant Genomics and Breeding Institute, Seoul National University, Seoul, Korea; 4 Bio-MAX Institute, Seoul National University, Seoul, Korea; 5 School of Applied Biosciences, Kyungpook National University, Daegu, Korea; Lawrence Berkeley National Laboratory, United States of America

## Abstract

Protein disulfide isomerase (PDI) is a chaperone protein involved in oxidative protein folding by acting as a catalyst and assisting folding in the endoplasmic reticulum (ER). A genome database search showed that rice contains 19 PDI-like genes. However, their functions are not clearly identified. This paper shows possible functions of rice PDI-like protein 1-1 (PDIL1-1) during seed development. Seeds of the T-DNA insertion *PDIL1-1* mutant, *PDIL1-1Δ,* identified by genomic DNA PCR and western blot analysis, display a chalky phenotype and a thick aleurone layer. Protein content per seed was significantly lower and free sugar content higher in *PDIL1-1Δ* mutant seeds than in the wild type. Proteomic analysis of *PDIL1-1Δ* mutant seeds showed that PDIL1-1 is post-translationally regulated, and its loss causes accumulation of many types of seed proteins including glucose/starch metabolism- and ROS (reactive oxygen species) scavenging-related proteins. In addition, PDIL1-1 strongly interacts with the cysteine protease OsCP1. Our data indicate that the opaque phenotype of *PDIL1-1Δ* mutant seeds results from production of irregular starch granules and protein body through loss of regulatory activity for various proteins involved in the synthesis of seed components.

## Introduction

Endosperm composition is an important component of cereal crops because starch is the major nutrient and source of carbohydrates. Rice endosperms contain major components of starch, and minor reserves, such as protein, lipid, and moisture. Because starch composes 80–90% of the total rice endosperm, the starch content affects the properties of the endosperm [1].

An important factor for determining starch texture is amylopectin and amylose contents. More amylopectin causes adhesive and opaque endosperms, as in seeds of glutinous rice [2]. In plants, amylopectin has a highly organized structure designated as a tandem-cluster structure, in which the structural unit clusters are linked in tandem to form an amylopectin molecule [3], [4]. The tandem-cluster structure of amylopectin is known to have an effect on the crystallization and properties of starch. In maize, starch synthase, starch branching enzyme, starch debranching enzyme, and ADP-glucose pyrophosphorylase are involved in the formation of the tandem-cluster structure of amylopectin, and mutants of these proteins resulted in opaque phenotypes [5]–[9]. Rice expressing a starch branching enzyme-deficient amylose-extender (*ae*) mutant had a floury endosperm that was composed of unusual branched glucans [10]. An isoamylase-lacking *sugary-1* mutant and other isoamylase-suppressing transgenic rice produced floury endosperms containing phytoglycogen, a highly branched structure of amylopectin [11]–[13]. A deficiency in rice pyruvate orthophosphate dikinase caused an opaque inner region in the endosperm due to disruption of the pyrophosphate-dependent pathway involved in starch metabolism [14].

To investigate the mechanism mediating the production of opaque endosperms, most studies have focused on starch synthesis-related enzymes. However, recent data report that other diverse factors are also associated with the opaque phenotype. For example, overexpression of a rice binding protein (BiP) produced an opaque phenotype seed with a floury endosperm [15]. In addition, maize starchy endosperm mutants showed increased expression of genes associated with unfolding protein response (UPR), a common feature of opaque mutants [16]. The UPR is critical for the maintenance of cellular function under endoplasmic reticulum (ER) stress, and BiP plays a pivotal role in the UPR [17], [18]. This UPR pathway is especially important in cereal crops because redundant storage proteins are synthesized in the endosperm, which is likely to be exposed under ER stress conditions. Thus, it seems that BiP controls endosperm development as a UPR regulator and chaperone in cereal crops.

Protein disulfide isomerase (PDI) functions in the synthesis and deposition of storage proteins. PDI is an abundant 57-kDa protein in eukaryotes, including yeast, mammals, and plants. It consists of four domains and an ER resident signal peptide, Lys-Asp-Glu-Leu (KDEL), at the C-terminus; two domains (a and a’) are homologous to thioredoxin and the two remaining domains (b and b’) are responsible for interacting with substrates [19], [20]. The a and a’ domains are active sites containing two cysteines in the sequence WCGHCK, whereas the b and b’ domains are not active sites. Because PDI has active thioredoxin-like domains and an ER resident signal, it assists folding by introducing proper disulfide bonds to nascent polypeptides in the ER lumen [21]. Several reports also suggest that PDI has chaperone activity and inhibits the aggregation of misfolded proteins that do not contain disulfide bonds [22]–[25].

PDI-like (PDIL) proteins are members of a multigene family within the thioredoxin (TRX) superfamily [26]. Houston et al. [27] identified multiple PDI-related genes by a genome-wide search and reported orthologous sets of 19 PDIL sequences in rice, and the authors termed these PDIL proteins because their biochemical functions had not been elucidated. The most prevalent and best-studied PDIL protein is an ortholog of an approximately 57-kD PDI that has been found in most eukaryotic organisms investigated, including plants [28], [32].

Several physiological roles for PDI have been reported in plants. At*PDI5*-deficient Arabidopsis mutant, *atpdi5,* showed premature initiation of program cell death (PCD) during seed development, indicating that PDI inhibits stimulation of PCD by blocking cysteine protease activity [29]. In rice, an *esp2* (endosperm storage protein 2) mutation caused loss of PDIL1-1 expression and accumulation of proglutelins, suggesting that PDIL1-1 retains glutelin precursors within the cisternal ER by chaperone activity, resulting in inhibition of abnormal aggregation of prolamin [30], [31]. In addition, PDIL proteins have different activities in rice. For example, PDIL1-1 facilitates the oxidative folding of vacuole-targeted storage proteins, such as proglutelins and α-globulin, whereas PDIL2-3 does not exert PDIL1-1 functions but promotes the specific localization of crP10 (Cys-rich 10-kD prolamin) in the core of PB-I (protein body-I) [32].

In this study, we analyzed *PDIL1-1* knock-out transgenic rice, and in particular, its seed. To characterize seed properties such as an opaque endosperm and a thick aleurone layer, the amount and composition of seed proteins were identified by proteomic methods. In addition, PDIL1-1 accumulation was investigated in developing seeds, and a PDIL1-1-interacting partner was isolated. Furthermore, inner seed factors such as starch granules, protein bodies, and free sugars were also examined. Here, we show that PDIL1-1 is a critical factor in seed development including in the development of the endosperm and aleurone layer through regulation of the proportion of various seed proteins including storage proteins.

## Materials and Methods

### Genotypic Analysis of T-DNA Insertion Knock-out Mutants of PDIL1-1

Two T-DNA insertion mutant rice, PFG_1B-16041 and PFG_2B-80111, were kindly provided by Dr. Gynheung An at POSTECH (Pohang, Korea). T-DNA insertion mutant rice was generated from the cultivar Dongjinbyeo (wild type) according to a method previously described [33], [34]. To identify T-DNA insertion sites within the *PDIL1-1* locus, primers were designed for T-DNA border sequences and the flanking region of the *PDIL1-1* (Table S1). The loci of inserted T-DNA in two mutant lines were identified by TAIL-PCR according to previously described methods [35]. The insertion sites were amplified by PCR using primers that specifically bind to the border sequence and flanking region of T-DNA. The same mutant lines were also examined by western blotting using an anti-PDIL1-1 antibody we previously generated [36]. Selected homozygote lines were used to produce progeny for this study.

### Two-dimensional Gel Electrophoresis and Imaging Analysis

Seed proteins were extracted from mature seeds of the wild type and *PDIL1-1Δ* mutant and separated by two-dimensional gel electrophoresis (2-DE). Sample preparation was performed by the following method. Whole seeds chilled in liquid nitrogen were homogenized using a mortar and pestle. The extraction buffer (7 M urea, 2 M thiourea, 4% (w/v) 3-[(3-cholamidopropy) dimethyammonio]-1-propanesulfonate (CHAPS), 1% (w/v) dithiothreitol (DTT), 2% (v/v) pharmalyte, and 1 mM benzamidine) was added to powdered samples and incubated for 1 h at room temperature. After centrifugation at 15,000 × *g* for 1 h at 15°C, the soluble fraction was withdrawn using 2-DE. The proteins were loaded onto an IPG dry strip equilibrated with another buffer (7 M urea, 2 M thiourea, 2% CHAPS, 1% DTT, and 1% pharmalyte). Isoelectric focusing was performed at 20°C using a Multiphor II electrophoresis unit and an EPS 3500 XL power supply (Amersham Biosciences) under the following conditions: linear increase from 150 to 3,500 V for 3 h; constant 3,500 V to reach 96 kV/h. Then, the strips were immersed for 10 min in solution (50 mM Tris-Cl (pH 6.8), 6 M urea, 2% SDS, and 30% glycerol) and transferred to SDS-polyacrylamide gels (20 × 24 cm, 10–16%). SDS-PAGE was carried out at 20°C with 1,700 V/h using a Hoefer DALT 2D system (Amersham Biosciences) according to the manufacturer’s instructions. After electrophoresis, gels were stained with silver nitrate as previously described [37]. From the stained gels, digitized images were acquired and quantitatively analyzed using the PDQuest (version 7.0, Bio-Rad) software. The quantity of each spot was normalized by total valid spot intensity. Protein spots to analyze with MALDI-TOF were selected based on a more than two-fold intensity increase or decrease as compared with control samples.

### Enzymatic Digestion of Proteins in Gels and MALDI-TOF Analysis

Protein spots were enzymatically digested in-gel using modified porcine trypsin as previously described [38]. Gel pieces were cleaned with 50% acetonitrile followed by rehydration with trypsin (8–10 ng/µL) and incubated for 8–10 h at 37°C. The reaction was terminated by addition of 5 µL of 0.5% trifluoroacetic acid. Tryptic peptides were combined from the aqueous phase of gel extractions with 50% aqueous acetonitrile through several rounds. After concentration, the peptides were desalted using C_18_ZipTips (Millipore, MA, USA) and eluted in 1–5 µL of acetonitrile. Purified peptides were mixed with an equal volume of α-cyano-4-hydroxycinnamic acid in 50% aqueous acetonitrile, and 1 µL was spotted onto a target plate. Protein analyses were performed using an Ettan MALDI-TOF (Amersham Biosciences). Peptides were evaporated with an N_2_ laser at 337 nm and accelerated with a 20 kV injection pulse for time-of-flight analysis. Each spectrum was a cumulative average of 300 laser shots. The database search program ProFound, developed by Rockefeller University (http://129.85.19.192/profound_bin/WebProFound.exe), was used to identify proteins by peptide mass fingerprinting. To determine the confidence of identified proteins, the Z-score, MASCOT score, and sequence coverage were used as the determining criteria. The proteins with a Z-score ≥1.28 were considered to be successfully identified (Z-score ≥1.28 indicates that the database match is nonrandom with a 90% probability). Proteins that were matched with a MASCOT score of greater than 64 were considered to be significant (*p*<0.05). The sequence coverage of matching peptides should not be less than 15%. Spectra were calibrated against trypsin auto-digestion ion peaks *m/z* (842.510, 2211.1046), which serve as internal standards.

### Measurement of Seed Protein Amount in Dry Seeds

Total protein amount was measured from husked grains using the Kjeldahl Protein/Nitrogen Analyzer. Husked rice grains of the wild type and *PDIL1-1Δ* mutant were milled using a mortar and digested for 90 min at 420°C with H_2_SO_4_ and Kjeltab Se (FOSS Tecator AB, Hoganas, Sweden) in a digestion apparatus (Digestor 2006; FOSS Tecator AB). After cooling, the sample was injected into a Kjeldahl analyzer (Kjeltec Auto 1035 Analyzer; FOSS Tecator AB). Distillation and titration were carried out automatically. Thereafter, total nitrogen content was measured, and the protein content was calculated by multiplying 6.25 as the protein conversion factor.

### Methylene Blue and Iodine Staining of Seeds

Whole or sectioned seeds were used for staining with methylene blue. Mature seeds of the wild type and *PDIL1-1Δ* mutant were cut with a razor blade. After immersion of the whole or sectioned seeds in 3% (v/v) triethanolamine for 30 s, the samples were stained with 0.05% (w/v) methylene blue and 0.025% (w/v) eosin Y for 1 min followed by washing with methanol briefly. The blue-stained aleurone layers were observed by optical microscopy (SJX7 Zoom Stereo Microscope; Olympus).

To investigate alterations in starch composition in seeds of the *PDIL1-1Δ* mutant, mature seeds of the wild type and *PDIL1-1Δ* mutant were cut with a razor blade and stained with iodine solution. Cross-sectioned seeds were immersed into 0.2% iodine and 2% potassium iodide solution and then observed by optical microscopy (SJX7 Zoom Stereo Microscope; Olympus).

### Measurement of Aleurone Layer Thickness

Mature seeds of the wild type and *PDIL1-1Δ* mutant were cross-sectioned with a razor blade, dried in a Critical Point Dryer (Balzers CPD 030; Bal-Tecn) and then analyzed with a scanning electron microscope (SEM, JSM-5410LV; JEOL). The aleurone layers were observed along with the edge of endosperm at ×150 magnification and the images were merged into a large picture for whole cross sections of the seeds using Photoshop CS3 software (Adobe Systems). The thickness of the aleurone layers was measured at intervals of 50 µm using the ImageJ softeware (NIH), and averages were calculated at three portions: dorsal, ventral, and lateral sides.

### Evaluation of PDIL1-1 Levels by Western Blot

To check PDIL1-1 in the *PDIL1-1Δ* mutant, wild type or *PDIL1-1Δ* mutant seeds were ground with mortar and pestle and total seed proteins were extracted with extraction buffer (50 mM Tris-Cl (pH 7.5), 150 mM NaCl, 1% Triton X-100, and 1 mM PMSF). After centrifugation at 12,000 × *g* for 15 min, supernatants were taken. Proteins were separated on 10% SDS (sodium dodecyl sulfate)-polyacrlyamide gel and then transferred onto PVDF (Polyvinylidene fluoride) membranes (Millipore). PDIL1-1 protein was detected by western blot with an anti-PDIL1-1 antibody [36] according to the manufacturer’s instructions (Pierce).

To investigate PDIL1-1 levels during seed development, total proteins were isolated from whole grains that were sampled at 5, 10, 20, 30, 40, and 50 days after flowering (DAF). After determining protein concentrations, equal amounts of the proteins were loaded and separated on a 10% SDS gel. After electrophoresis, the proteins were transferred onto PVDF membrane, and detected as above.

To check the PDIL1-1 distribution in the outer part of endosperm and aleurone layers, differently polished grains were prepared by gradually polishing the whole grains of the wild type using polisher with reducing 1% weight. Differently husked grains were milled with liquid nitrogen and proteins were analyzed by western blot with an anti-PDIL1-1 antibody [36] according to the manufacturer’s instructions.

### Quantitative Real-time RT-PCR

Total RNA was extracted from different developmental stages and tissues to investigate spatial and temporal expression patterns of *PDIL1-1* according to a method previously described [39]. Total RNA was quantified into equal concentrations, and first-strand cDNA was synthesized with 5 µg of total RNA using the iScript™ cDNA Synthesis Kit (Bio-Rad). Then, an equal volume of cDNA was amplified by quantitative real-time PCR (MyiQ, Bio-Rad) according to the manufacturer’s protocol. The specific primers and template cDNA were combined with 25 µL iQ™ SYBR® Green Super Mix (Bio-Rad), and reactions were performed by the following thermal conditions: 50°C for 2 min; 95°C for 10 min; and 40 cycles of 95°C for 15 sec and 60°C for 1 min. The C_T_ values of target genes were normalized to the C_T_ value of *OsActin* (Accession No. X16280) and analyzed with iCycler IQ™ software (Bio-Rad). RT-PCR primers were designed using Primer3 (http://frodo.wi.mit.edu/cgi-bin/primer3/primer3.cgi), and their specificity was verified by cloning into the pGEM T-Easy vector (Promega) and sequencing with an ABI 3730xl DNA Analyzer (Applied Biosystems). The primer sets used for these studies are listed in Table S2.

### Northern Blot Analysis

Total RNA was isolated from whole grains that were sampled at 5, 10, 20, 30, 40, and 50 DAF as previously described [35]. Total RNA was separated by formaldehyde-agarose gel electrophoresis and transferred onto a nylon membrane (Millipore). The membrane was hybridized with α-^32^P-labeled *PDIL1-1* cDNA followed by washing several times to remove non-specifically bound probe and exposed on X-ray film at −70°C.

### Observation of Starch Granules and Protein Body

To observe starch granules on the surface of transversely sectioned endosperm, mature seeds of the wild type and *PDIL1-1Δ* mutant were cut with a razor blade. Sliced specimens were fixed in PFA (2% paraformaldehyde, 2% glutaraldehyde, and 50 mM sodium phosphate buffer (pH 7.2)), and then washed with 50 mM sodium cacodylate buffer (pH 7.2) three times. After fixation, the specimens were dehydrated in gradually increasing concentrations of ethanol, followed by drying with 100% isoamyl acetate in a Critical Point Dryer (Balzers CPD 030; Bal-Tec). The prepared specimens were mounted on metal stubs for coating with gold particles and moved to a scanning electron microscope (SEM, JSM-5410LV; JEOL). To observe starch granule morphology, mature seeds of the wild type and *PDIL1-1Δ* mutant were powdered with a grinder (Grinder A11; IKA). The powder was extracted with buffer (55 mM Tris-Cl (pH 6.8), 2.3% SDS, 10% glycerol, and 5% 2-mercaptoethanol), and then centrifuged at 2,500 × *g* for 10 min. The samples were washed with distilled water and methanol, and then filtered through a 40 µm mesh filter. Purified starch was dried in air, coated with gold particles, and then finally observed by SEM.

Protein bodies were observed by transmission electron microscopy (TEM). The cross-sectioned seeds of the WT and *PDIL1-1Δ* mutant were fixed for 2 h in 2% paraformaldehyde and 2% glutaraldehyde in 50 mM sodium cacodylate buffer (pH 7.2). The samples were washed in 50 mM sodium cacodylate buffer and then distilled water and then stained with 0.5% uranyl acetate at 4°C overnight. Dehydration of the samples was conducted by a series of ethanol concentrations, and samples were infiltrated with propylene oxide and Spurr’s resin before polymerization. The embedded samples were sectioned with an Ultramicrotome (RMC MT-X; Beockeler Industries) and then stained with 2% uranyl acetate and Reynold’s lead citrate before TEM observation.

### Determination of Starch, Amylose, Free Sugar, and Lipid Contents

Starch content was measured using the Starch Assay Kit (SA-20; Sigma) according to the manufacturer’s protocols. Powdered seed flours of the wild type and *PDIL1-1Δ* mutant were weighed to 0.1 mg accuracy, and then purified starch from the sample was solubilized by the DMSO/HCl method. Amylose contents in wild type and *PDIL1-1Δ* mutant seeds were determined as previously described [40]. For measurement of lipid content, total lipid was extracted from wild type and *PDIL1-1Δ* mutant seeds with diethyl ether, and the contents were analyzed by the Soxhlet method (Soxtec 2043 System; FOSS Tecator AB).

For analysis of carbohydrate, wild type and *PDIL1-1Δ* mutant seeds were homogenized using liquid nitrogen, and then powder was mixed with distilled water for 24 h. After centrifuge, supernatant was filtered with a 0.25 µm mesh, and then diluted extracts was injected into a HPLC system (HP1100 series LC/MSD; HP). The mobile phase was used with acetonitrile and water at a flow rate of 2 mL/min and the tube temperature was maintained at 85°C. Eluted carbohydrates were monitored by an evaporative light scattering detector (ELSD 2420, Waters).

### X-ray Diffraction Analysis

The X-ray diffraction patterns were analyzed as previously described [41]. Husked seeds of WT and the *PDIL1-1Δ* mutants were crushed using a grinder (Grinder A11; IKA, China) and filtered with 0.45 µm mesh. In order to separate the outer and inner endosperm, de-embryonated dry seeds were polished to 10% weight loss and the powders were collected. The remaining seeds were also crushed, used and filtered as described above.

Then, the samples were equilibrated in a 100% relative humidity chamber for 24 h at room temperature. The X-ray diffraction patterns were obtained with copper, nickel foil-filtered, Kα radiation using a Powder X-Ray Diffractometer (D5005; Bruker) at 40 kV and 40 mA. The scanning region of the two-theta angle (2θ) ranged from 6.0 to 40.0° with a scan speed of 1 deg/min.

### Isolation of OsCP1 by Yeast Two-hybrid

The Arabidopsis cDNA library generated from mRNA isolated from 3-day-old etiolated wild-type (Columbia) seedlings was used to isolate PDIL1-1-interacting proteins. To do this, Arabidopsis cDNAs were cloned into the pACT vector (Clontech) containing a GAL4 activating domain and then introduced into AH109 yeast cells. Next, full-length *PDIL1-1* cDNA was cloned into the pGBKT7 vector (Clontech) containing a GAL4 binding domain, introduced into the transformed AH109 yeast cells, and then screened on synthetic media lacking leucine and tryptophan. Selected clones were rescreened on media lacking leucine, tryptophan, and histidine with 5 mM 3-amino-1,2,4-triazole (3-AT). Plasmids were rescued from the selected cells, and inserted DNAs were identified by DNA sequencing (ABI 3730 XL DNA analyzer, Applied Biosystems). We identified that CP43, a papain-like cysteine protease, interacts with PDIL1-1. Next, to isolate CP43 in rice, we amplified its rice homologs, oryzain α, oryzain β, papain-like cysteine protease XBCP3, and cysteine protease CP1 (OsCP1) cDNAs by RT-PCR. The oligonucleotides for RT-PCR were as follows: forward, 5'-ATGAGGATTTCCATGGCTCTC-3', and reverse, 5'-TCAAGCGCTGCTCTTCTTG-3', for oryzain α amplification; forward, 5'-ATGGCCGCCCGCGCCGCC-3', and reverse, 5'-TCATGCGGTGTTCAGCTT-3', for oryzain β amplification; forward, 5'-ATGGCGTTCGTCTCCTGC-3', and reverse, 5'-TCACTGATCCAACAGTTC-3', for XBCP3 amplification; and forward, 5'-ATGGCAGGCGGTGGCGGCAA-3', and reverse, 5'-CTATACTAGATCTTCCCT-3', for OsCP1 amplification. PCR products were cloned into the pGADT7 vector (Clontech) containing a GAL4-activating domain. After co-introduction with pGBKT7-PDIL1-1 and pGADT7-OsCP1 into AH109 yeast cells, protein interaction was examined as described above.

### Phylogenetic Analysis

The protein sequences of cysteine proteases that were homologous to PDIL1-1 were aligned by the CLUSTALW2 program (http://www.ebi.ac.uk/Tools/msa/clustalw2/) and then manually adjusted to minimize gaps by using MEGA5 software [42]. A phylogenetic tree was generated by the neighbor-joining method with 1,000 bootstrap replicates using MEGA5 software.

## Results

### Isolation of *PDIL1-1Δ* Mutant Rice and Phenotypic Characterization of its Mutant Seeds

**Figure 1:**
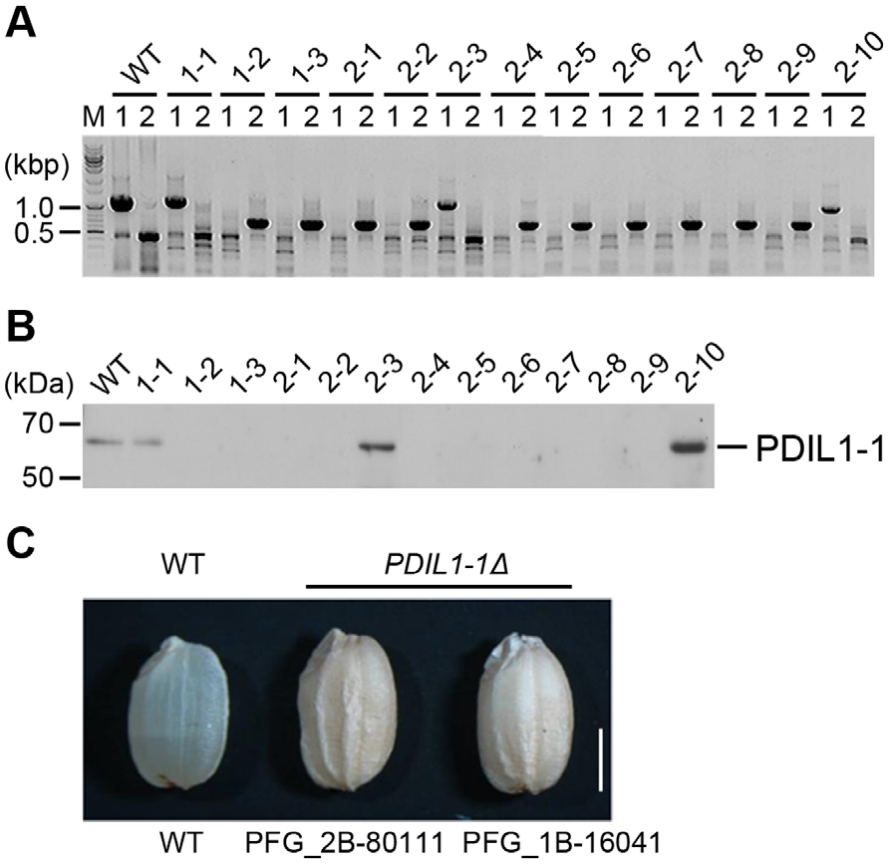
Isolation of the *PDIL1-1Δ* mutant and its seed phenotype. (A) Identification of the T-DNA insertion site in the *PDIL1-1* gene (locus number Os11g09280) by PCR. T-DNA insertion mutant PFG_1B-16041.R was provided by Dr. Gynheung An, POSTECH. Independent transgenic lines were analyzed by PCR using two sets of primers (described in Table S1); 1,229-bp fragments were amplified by PCR with LP and RP, but not by the primer set in transgenic homozygote lines. Approximately 700-bp fragments were amplified by PCR with BP and RP. (B) Identification of *PDIL1-1* mutant rice by western blot. Total seed proteins extracted from the lines described in (A) were separated by SDS-PAGE and examined by western blot using an anti-PDIL1-1 antibody. (C) Grains of wild type and *PDIL1-1Δ* mutants were collected, and palea and lemma of the grains of wild type and two *PDIL1-1Δ* mutant alleles (PFG_1B-16041.R and PFG_2B-80111.R) were opened and removed. The mutants showed a chalky and uneven phenotype. Bar, 0.3 cm.

In eukaryotic cells, most proteins are post-translationally modified after synthesis in the ER, with the formation and isomerization of disulfide bonds an important occurrence after translation. To investigate the roles of PDIL1-1 during seed development, we isolated two rice T-DNA insertion mutants, PFG_1B-16041 and PFG_2B-80111, by TAIL-PCR and western blotting (Figure 1A and B, Figure S1A and B). T-DNA was located between amino acid residues 450 and 451 within the tenth exon in PFG_1B-16041 and between the first and second exon corresponding to the first intron in PFG_2B-80111 (Figure S2A and B).

We next examined the *PDIL1-1Δ* phenotype by visual inspection and microscopy. *PDIL1-1Δ* plants displayed a similar phenotype to that of the wild type. However, a difference was noted between wild type and *PDIL1-1Δ* mutant seeds. The dry weights of the seeds were lower in *PDIL1-1Δ* compared to the wild type, which is in agreement with a recent report [43]. The most noticeable feature of the *PDIL1-1Δ* mutant seeds were shrunken and floury phenotypes (Figure 1C) as previously shown [43]. We chose line number 2–1 (one of the homozygote lines of PFG_1B-16041 mutants, Figure 1A) for further experiments and named it *PDIL1-1Δ*.

### Various Seed Proteins Accumulated in the *PDIL1-1Δ* Mutant

PDI can control the stability and activity of target proteins by regulating formation and isomerization of disulfide bonds. Therefore, we examined the total protein content in endosperms of the *PDIL1-1Δ* mutant. We found that the total protein content per seed was much lower, whereas total seed protein content per equal seed weight was up to 114% higher in the *PDIL1-1Δ* mutant (Table 1) compared to the wild type.

**Table 1:** Comparison of seed weight and protein amounts between WT and *PDIL1-1Δ* mutant.

	Seed weight (mg)	Protein (mg)/grain	Protein (mg)/dry weight (g)
WT	25.8±0.06	1.86±0.02	72.04±0.94
*PDIL1-1Δ*	17.2±0.62^**^	1.41±0.10*	82.02±6.07^**^

Palea and lemma of the grains of the wild type and *PDIL1-1Δ* mutant (0% milled rice) were removed and then used for analysis of seed weight, and protein amounts.

The numerical values represent the mean of three independent experiments, and the values are expressed as means ± standard deviations.

* and **indicate significant difference from the wild type at P<0.05 and P<0.01 by t-test, respectively.

**Figure 2:**
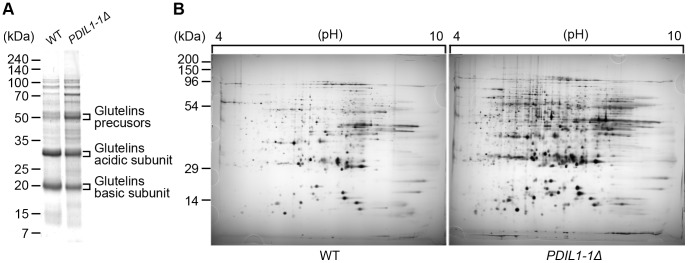
Comparison of seed proteins of WT and *PDIL1-1Δ* mutant. (A) Total seed proteins were extracted from the WT and *PDIL1-1Δ* mutant and separated by 10–27% gradient SDS-PAGE. (B) Total proteins were extracted from mature seeds of the WT and *PDIL1-1Δ* mutant and then analyzed by 2-DE.

Therefore, we analyzed mature seed proteins of the *PDIL1-1Δ* mutant. One-dimensional gel analysis showed that the glutelin precursor protein accumulated in the *PDIL1-1Δ* mutant (Figure 2A), supporting previous results [30]. A two-dimensional gel assay revealed that the levels of 84 proteins were up-regulated in the *PDIL1-1Δ* mutant compared to the wild type. Among them, the expression level of 36 proteins increased by more than two-fold in the *PDIL1-1Δ* mutant compared to the wild type (Figure 2B). Interestingly, only sucrose synthase GT1 was highly up-regulated in the wild type. We identified 36 protein spots by MALDI-TOF/MS (Table 2). The results showed that these proteins are involved in post-translational modification, pathogen defense, glucose/starch metabolism, stress resistance, amino acid synthesis, reactive oxygen species (ROS) scavenging, and signal transduction [37], [38].

**Table 2:** List of proteins for which spot intensity changed over two-fold in *PDIL1-1Δ* seeds compared to WT.

						1st		2nd	
Spot No.	Protein funtions	Accession number	Homologous protein	PI	MR	WT	*OsPDIL1-1Δ*	WT	*OsPDIL1-1Δ*
1307R	Amino acid metabolism	NP_001047033	Nitrilase/cyanide hydratase and apolipoprotein N-acyltransferase domain containing protein	4.73	39.38	37.66	383.22	183.53	367.42
6511R	Amino acid metabolism	NP_001060945	Tryptophan synthase beta chain 1	6.07	49.30	54.16	302.28	ND	276.32
6808R	Amino acid metabolism	NP_001067314	Ethylene-responsive methionine synthase	6.06	88.82	432.64	1108.8	29.53	1859.3
7501R	Amino acid metabolism	AAK52114	Putative alanine aminotransferase	6.27	53.95	372.62	773.71	151.1	457.03
0007R	Carbohydrate metabolism	NP_001049720	Glyoxalase/bleomycin resistance protein/dioxygenase domain containing protein	4.52	11.45	123.87	407.61	55.9	510.18
1003R	Carbohydrate metabolism	NP_001049720	Glyoxalase/bleomycin resistance protein/dioxygenase domain containing protein	4.61	11.51	ND	2105.78	74.28	1374.67
3203R	Carbohydrate metabolism	NP_001063741	Glucosamine/galactosamine-6-phosphate isomerase domain containing protein	5.09	32.06	717.21	1891.84	427.35	1575.02
4402R	Carbohydrate metabolism	NP_001046020	Phosphoglycerate kinase, cytosolic	5.23	46.15	ND	485.15	ND	817.02
4406R	Carbohydrate metabolism	NP_001046020	Phosphoglycerate kinase, cytosolic	5.26	46.18	291.09	1420.95	185.22	1231.2
4413R	Carbohydrate metabolism	NP_001046020	Phosphoglycerate kinase, cytosolic	5.36	46.16	448.33	1351.23	495.86	1586.28
4520R	Carbohydrate metabolism	NP_001046020	Phosphoglycerate kinase, cytosolic	5.23	47.39	ND	866.06	ND	1311.58
5409R	Carbohydrate metabolism	NP_001046020	Phosphoglycerate kinase, cytosolic	5.54	47.58	35.76	555.44	ND	885.41
6502R	Carbohydrate metabolism	NP_001056586	Cytosolic 6-phosphogluconate dehydrogenase	5.60	52.79	627.47	1791.62	152.43	841.81
7207R	Carbohydrate metabolism	NP_001041764	Ricin B-related lectin domain containing protein	7.02	36.04	ND	380.32	ND	564.36
7807R	Carbohydrate metabolism	AAC41682	GT1_Sucrose_synthase	6.99	94.91	970.01	89.88	540.53	ND
7101R	Nucleotide metabolism	EAY80855	P-loop containing Nucleoside Triphosphate Hydrolases	6.30	22.84	184.51	379.05	72.05	1067.83
7003R	Nucleotide metabolism	NP_001056515	Nucleoside diphosphate kinase III	6.79	12.90	ND	561.81	ND	2397.5
8003R	Nucleotide metabolism	NP_001056515	Nucleoside diphosphate kinase III	7.56	13.94	ND	1312.84	ND	1405.59
8009R	Nucleotide metabolism	NP_001056515	Nucleoside diphosphate kinase III	7.74	13.89	ND	259.79	144.29	821.77
0004R	Oxidative stress	NP_001059069	Thioredoxin H-typeTRX-H	4.30	10.62	ND	954.26	77.06	483.89
3005R	Oxidative stress	ABR25528	peroxiredoxin 5 cell rescue, defense and virulence	5.06	14.89	ND	674.08	ND	245.58
4102R	Oxidative stress	NP_001055195	Iron/manganese superoxide dismutases	5.20	25.43	260.01	2331.78	734.61	2578.43
5111R	Oxidative stress	NP_001060407	Peroxiredoxin 6	5.52	27.51	237.63	1537.77	891.33	2733.12
6604R	Oxidative stress	NP_001054699.1	Mercuric reductase family protein	5.78	55.36	148.11	490.95	88.09	394.51
1705R	Posttranslational regulation	AAB63469	endosperm lumenal binding protein	4.65	81.98	658.21	3642.48	746.09	2416.03
3107R	Signal transduction	EEC79379	PhosphatidylEthanolamine-Binding Protein	5.15	19.61	ND	2270.88	ND	1709.72
8201R	Signal transduction	NP_001048149	Annexin	7.23	35.93	136.65	1111.2	170.08	1099.36
8313R	Signal transduction	NP_001048149	Annexin	7.99	37.82	ND	1493	842.07	1799.13
0006R	Pathogen defence	NP_001042976	Jacalin-like lectin domain	4.42	11.15	ND	363	ND	337.09
5103R	Pathogen defence	NP_001049857	Pathogen-related proteinJIOsPR10	5.47	15.53	228.26	5425.54	251.21	3393.08
5502R		NP_001042239	Transferase family protein	5.40	49.83	805.1	1710.69	587.18	1344.15
1002R		EAZ06941	hypothetical protein	4.58	13.47	39.1	1353.84	317.26	756.18
5105R		BAD36320	hypothetical protein	5.48	16.88	274.48	1365.86	546.76	1762.57
5204R		NP_001044131	hypothetical protein	5.46	33.24	ND	780.41	ND	577.29
4202R		AAN05517	unknown protein	5.20	35.83	ND	187.67	ND	449.94
4203R		AAN05517	unknown protein	5.20	36.74	ND	588.13	ND	915.52

This experiment was repeated three times using different WT and the *PDIL1-1Δ* mutant seeds. Here, two results are shown.

Accession numbers indicate GenBank accession number.

ND indicate ‘not detectable’.

Next, to determine if any correlation exists between protein content and protein body or structure, we checked protein bodies in the sectioned endosperm by TEM. The results showed that irregular PB-I was found in the gaps between the starch granules in the endosperms of the *PDIL1-1Δ* mutant (Figure S3) as shown in the *esp2* mutant [30].

### *PDIL1-1Δ* has a Thick Aleurone Layer

**Figure 3:**
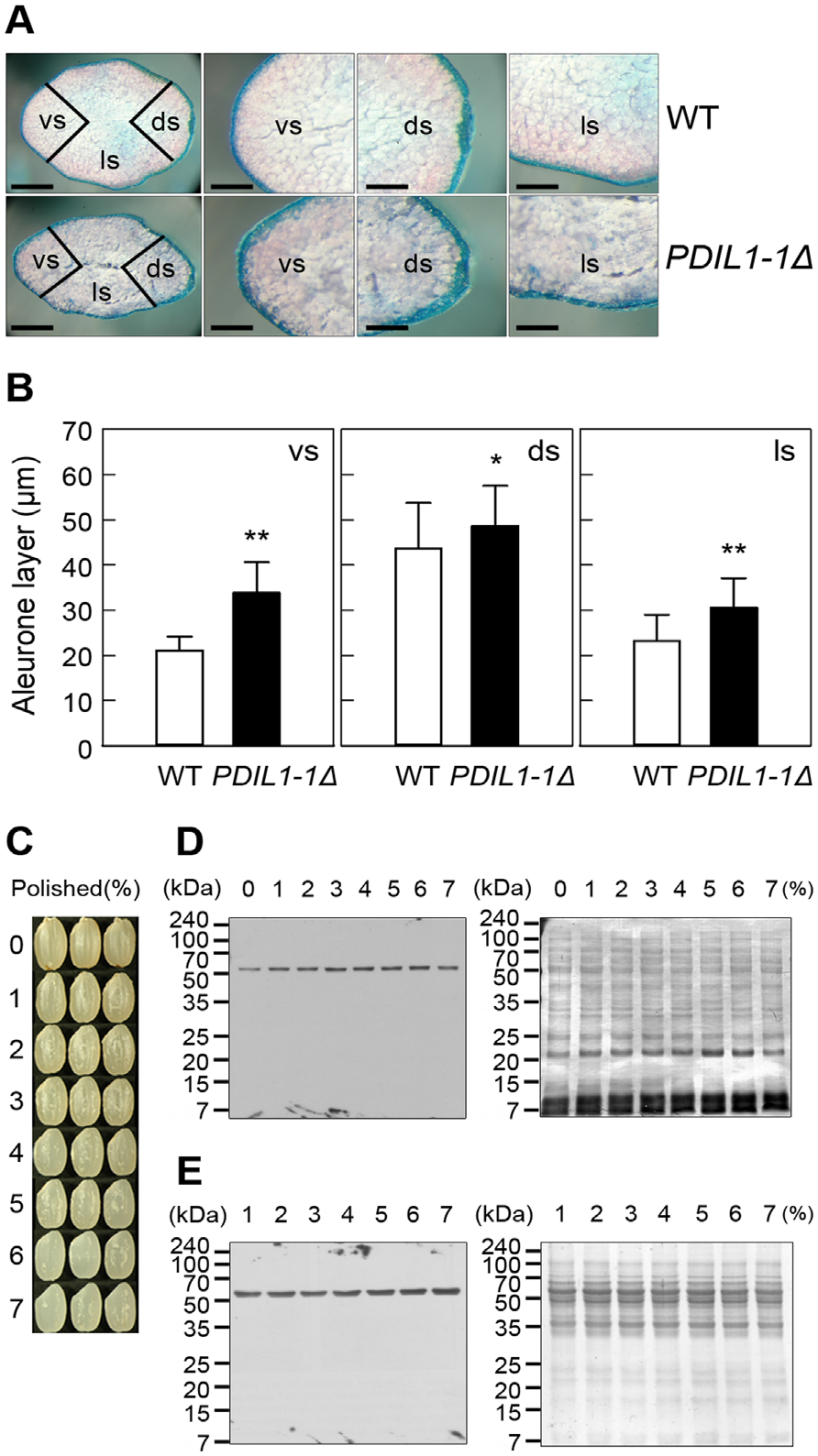
Aleurone layers of *PDIL1-1Δ* mutant seeds and analysis of PDIL1-1 protein level in differently polished seeds. (A) Mature seeds of the WT and *PDIL1-1Δ* mutant were stained with methylene blue and then analyzed by light microscopy. Abbreviations: vs, ventral side; ds, dorsal side. ls, lateral side. Bar, 1 mm (left), and 0.5 mm (middle and right). (B) Thickness of aleurone layers was measured in both seeds of the WT and *PDIL1-1Δ* mutant. * and ** indicate a significant difference from the wild type at P<0.05 and P<0.01 by t-test, respectively. (C) Dried seeds were polished to different extents. (D The remaining seeds were ground thoroughly, and the PDIL1-1 level was examined by western blot with an anti-PDIL1-1 antibody (left). After detection, the membrane was stained with Coomassie brilliant blue (right). (E) PDIL1-1 levels were examined in the polished powder by western blot with an anti-PDIL1-1 antibody (left). After detection, the membrane was stained with Coomassie brilliant blue (right).

The shrunken phenotype of the *PDIL1-1Δ* mutant seed led us to investigate the thickness of the aleurone layer of the *PDIL1-1Δ* mutant seed by TEM. We examined the aleurone layer by light microscopy after staining with methylane blue, revealing a significantly thicker aleurone layer in the *PDIL1-1Δ* mutant seeds than in the wild type (Figure 3A). We also measured the thickness of the aleurone layer by electron microscopy. Results showed that the ventral and lateral sides were significantly thicker and the dorsal side was slightly thicker in the *PDIL1-1Δ* mutant seeds compared to the WT (Figure 3B), suggesting that PDIL1-1 may be involved in the development of the aleurone layer or outer part of the endosperm. Thus, to evaluate the effect of PDIL1-1 on aleurone layer development, we investigated the PDIL1-1 distribution in the outer part of the endosperm including the aleurone layer. This was done by western blot analysis using differently husked and milled grains (Figure 3C). The results showed that PDIL1-1 levels were not significantly different in all the tested regions including the aleurone layer (Figure 3D and E), indicating that PDIL1-1 was evenly distributed in the endosperm and that the shrunken phenotype of the *PDIL1-1Δ* mutant seed was not a direct effect of the amount of PDIL1-1.

### Content of Free Sugars was Altered in the *PDIL1-1Δ* Mutant

**Figure 4:**
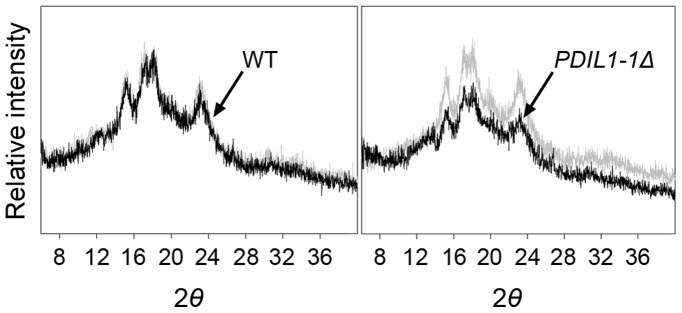
X-ray diffraction analysis of outer endosperm of the *PDIL1-1Δ* mutant seeds. After polishing of the WT and the *PDIL1-1Δ* mutant seeds to a 10% weight loss, powders (the outer part of the endosperm), were collected and analyzed by an X-ray diffractometry. The two-theta angle (2θ) ranging from 4.0 to 40.0° was scanned to obtain values that were overlapping for comparison of the two samples.

We next examined the floury phenotypes of the *PDIL1-1Δ* mutant using sectioned seeds of the *PDIL1-1Δ* mutant. Transversely or vertically sectioned seeds of the *PDIL1-1Δ* mutant on an illuminator revealed a much a darker color compared to the wild type (Figure S4A). Examination of the same seeds using optical microscopy showed an opaque endosperm, particularly in the outer part of the endosperm (Figure S4B), which is consistent with recently published data [43]. Because starch is a major component of the endosperm of rice, the phenotypes of the *PDIL1-1Δ* mutant seeds must be caused by alterations in the composition of starch or by a disorder of packing of starch granules during seed development stages. Previous results showed that the endosperm of the rice *sugary-1* mutant (EM-914) remained white after staining with I2-KI solution, whereas other mutant types such as EM-935 turned blue-black in non-waxy rice [12]. Thus, to determine the starch type, we examined the endosperm of the *PDIL1-1Δ* mutant seeds by staining with iodine solution and analyzing the X-ray powder diffraction patterns. Iodine staining analysis showed that the endosperms of the wild type and the *PDIL1-1Δ* mutant plants turned blue-black (Figure S5). Furthermore, X-ray diffraction analysis revealed nearly the same diffraction area and peak pattern (Figure S6). Therefore, to uncover the reason for the opacity of the *PDIL1-1Δ* mutant seed endosperm, we examined the sectioned seeds of *PDIL1-1Δ* by SEM. Interestingly, the results showed that the endosperms of *PDIL1-1Δ* mutant seeds had round starch granules (Figure S7) as recently reported [43]. In addition, this opaque phenotype of *PDIL1-1Δ* mutant seeds is much clearer in the outer part of the endosperm (Figure S4B) than in the inner part. Thus, the outer part of the endosperm was again examined by X-ray diffraction analysis. The results showed a low diffraction area in *PDIL1-1Δ* mutant seeds although peak patterns were similar for both WT and *PDIL1-1Δ* mutant seeds (Figure 4), which may be caused by an amorphous starch structure that was not crystallized because of round starch granules due to the change of starch composition in the outer endosperm of the *PDIL1-1Δ* mutant seeds.

Previous reports show that the endosperms of several mutants have opaque phenotypes, which are caused by alterations in starch and amylose contents [14], [44], [45]. We thus investigated the starch and amylose contents in the endosperms of *PDIL1-1Δ* mutant seeds. The result showed that the amount of starch and amylose in endosperm of the *PDIL1-1Δ* mutant were similar, or slightly higher as recently reported, to that of the wild type (Table S3) [43]. However, the content of free sugars such as sucrose, glucose, and maltose per weight was significantly higher in the endosperm of the *PDIL1-1Δ* mutant compared to that of the wild type (Table 3). By contrast, lipid level was similar, or slightly higher as recently reported, in the endosperms of the *PDIL1-1Δ* mutant and wild-type endosperms (Table S3) [43].

**Table 3:** Free sugar content in *PDIL1-1Δ* mutant seeds.

	WT (mg/dry weight (g))	*PDIL1-1Δ* (mg/dry weight (g))
Sucrose	6.51±0.30	9.07±0.30**
Glucose	9.87±2.29	11.36±1.03^NS^
Fructose	1.37±0.02	2.76±0.16**

Palea and lemma of the grains of the wild type and *PDIL1-1Δ* mutant (0% milled rice) were removed and used for analysis of free sugar content.

The numerical values represent the mean of three independent experiments, and the values are expressed as means ± standard deviations.

** indicates a significant difference from the wild type at P<0.01 and NS indicates no significant difference by t-test.

### The *PDIL1-1* Gene is Expressed in Various Tissues Including Immature and Mature Seeds

**Figure 5:**
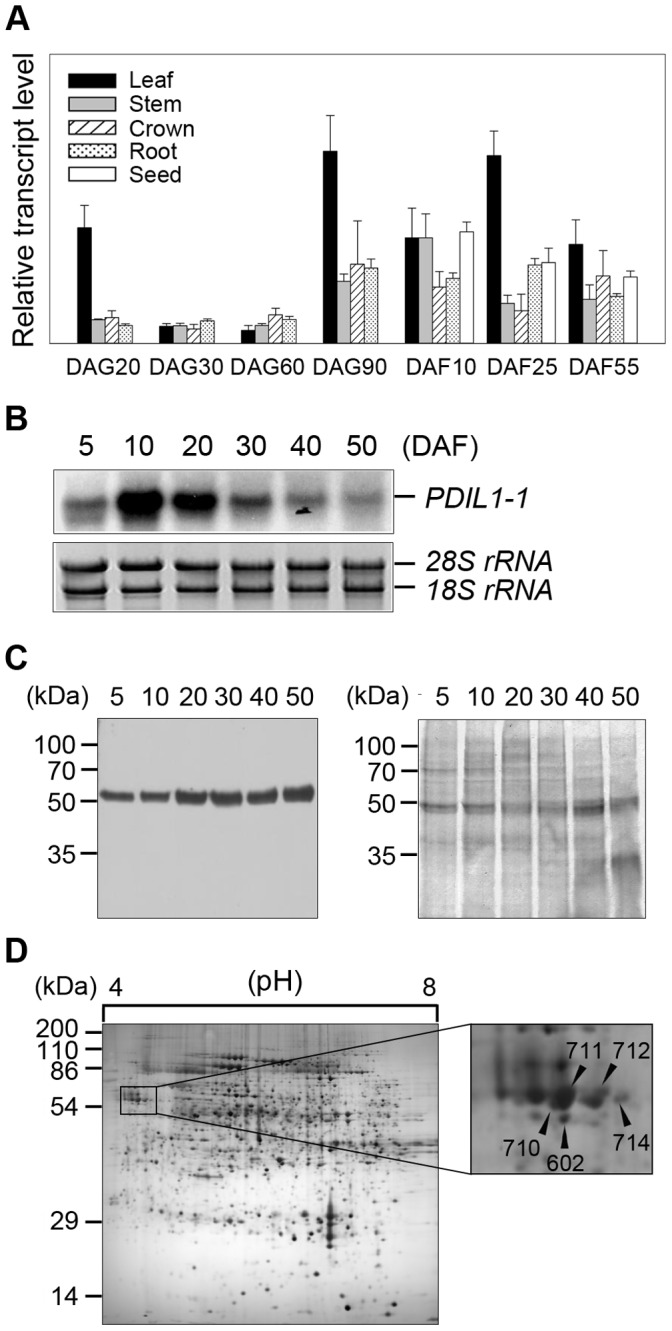
Expression pattern of *PDIL1-1* gene. (A) Expression profile of *PDIL1-1 I* gene during development. Total RNA was isolated from developing rice organs at the indicated time points and used for real-time RT-PCR. DAG, day after germination; DAF, days after flowering. (B) Total RNA was isolated from developing seeds at the indicated time points and separated on formaldehyde-agarose gels. After electrophoresis, total RNA was transferred onto a nylon membrane. The membrane was hybridized with ^32^P-labeled *PDIL1-1* cDNA and exposed on X-ray film. DAF, days after flowering. (C) Examination of the level of PDIL1-1 protein during seed development. Total proteins were extracted from the wild type at the indicated time points. After 12% SDS-PAGE, proteins were transferred onto a nitrocellulose membrane and treated with an anti-PDIL1-1 antibody (left). After blotting, the membrane was stained with Coomassie brilliant blue (right). (D) Proteomic analysis of rice seed proteins. Total proteins were extracted from mature seeds of WT and then separated by 2-DE. Each protein spot was identified by MALDI-TOF MS (left). The boxed region was enlarged (right). Arrowheads indicate PDIL1-1 protein spots.

To understand the possible regulatory roles of PDIL1-1 protein during rice development, we investigated expression of the *PDIL1-1* gene in leaf, root, stem, crown, and seed. Transcript levels were examined by real-time RT-PCR using total RNA isolated from specific tissues at specific stages during development. The results showed that *PDIL1-1* was expressed in all tissues throughout mature developmental stages, although with varying levels of expression (Figure 5A). Next, we checked the *PDIL1-1* transcript levels during seed development in detail. Northern blot analysis showed that *PDIL1-1* transcript levels were highest at 10 DAF and rapidly decreased to basal levels, with low expression seen even at 50 DAF (Figure 5B). However, PDIL1-1 protein levels steadily increased until 50 DAF during seed development (Figure 5C). Thus, to investigate whether PDIL1-1 stability or activity is post-translationally regulated, we performed 2-DE and identified protein spots with mass spectrometry. The results showed that five different protein spots were identified as PDIL1-1 (Figure 5D, Table S4), suggesting that its stability or activity can be regulated by post-translational modification or processing.

### PDIL1-1 Interacts with the Cysteine Protease OsCP1

**Figure 6:**
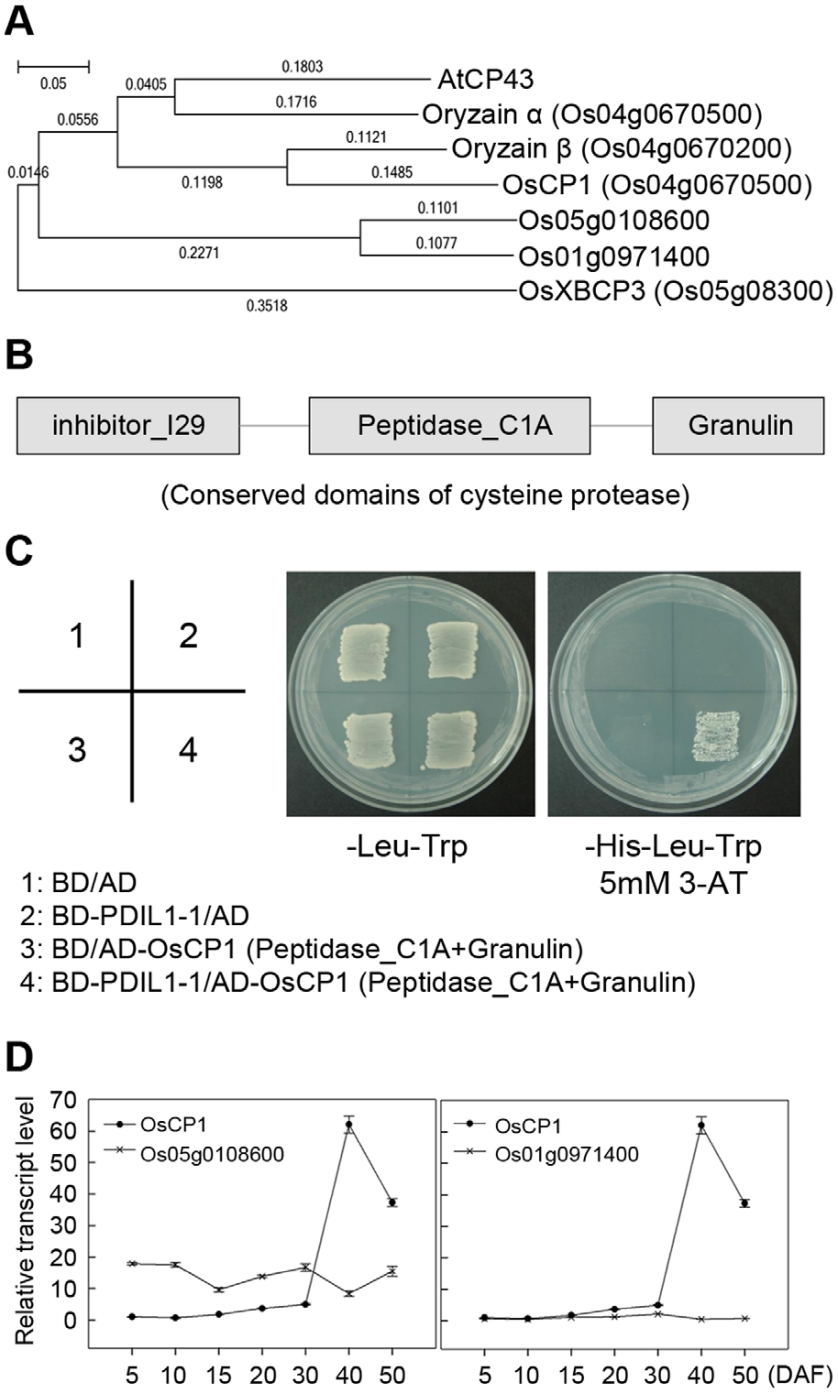
Physical interaction of PDIL1-1 with OsCP1. (A) Phylogenetic tree of *Arabidopsis* cysteine protease 43 (AtCP43) amino acid sequences with its rice homologs. The sequences of the proteins were aligned using the CLUSTALW2 software program and a phylogenetic tree was generated using the MEGA5 software program. B) Map of conserved domains of OsCP1. The three conserved domains, inhibitor_I29, peptidase_C1A, and granulin, are indicated by boxes. (C) Partial *OsCP1* cDNA encoding peptidase_C1A and granulin, and full-length *PDIL1-1* cDNA were fused to sequences encoding the Gal4 activation domain (AD) and the Gal4 DNA-binding domain (BD) in pGADT7 and pGBKT7, respectively. Each number indicates yeast cells transformed with a combination of only pGADT7 or pGBKT7 vectors or recombinant plasmids. Combinations are described in the box. Transformants were plated onto minimal medium (Leu^−^/Trp^−^) and (Leu^−^/Trp/His^−^ (5 mM 3-AT)), incubated for 4 days, and then photographed. (D) Expression analysis of *OsCP1* and its homologs. Transcript levels of *OsCP1* and its homologs, Os05g0108600 and Os01g0971400, were examined by real-time RT-PCR with gene-specific primers. These experiments were repeated three times independently. The reported values of transcript levels of *OsCP1* homologs are normalized to numerical values relative to the transcript level of *OsCP1* in DAF5, which is set at a value of 1.00±0.00. DAF, day after flowering.

Accumulating data suggest that PDIL1-1 protein can regulate many interacting partners. In addition, PDIL1-1 stability and activity may be regulated by other proteins. Thus, to isolate PDIL1-1′s upstream or target and downstream proteins, we first screened an Arabidopsis cDNA library by yeast two-hybrid analysis. We used an Arabidopsis library because a larger number of proteins are characterized in Arabidopsis compared to rice, which facilitates characterization of the function of the isolated proteins in rice. As a result, we found that the papain-like cysteine protease, CP43, strongly interacts with PDIL1-1 (data not shown). Next, we tried to isolate its homolog in rice. First, we checked the rice homologs by phylogenetic analysis. We found that oryzain α, oryzain β, and cysteine protease CP1 are evolutionarily very close to *Arabidopsis* CP43, whereas papain-like cysteine protease XBCP3 is relatively far from it on the phylogenetic tree (Figure 6A). Thus, we screened the rice CP43 homologs oryzain α, oryzain β, XBCP3, and CP1 by yeast two-hybrid analysis to determine if they interact with PDIL1-1. The results showed that PDIL1-1 can only interact with the cysteine protease CP1 (OsCP1) (Figure 6B and C). However, as shown in the phylogenetic tree, there are still two other OsCP1 homologs that remained unidentified, Os05g0108600 and Os01g0971400. Thus, we examined their expression levels and patterns by real-time RT-PCR during seed development. This revealed that *OsCP1* is relatively highly expressed compared with Os05g0108600 and Os01g0971400 during seed maturation, although the expression of Os05g0108600 is slightly high at early stages of seed development (Figure 6D). These results suggest that OsCP1 protease activity can be regulated by PDIL1-1, or that PDIL1-1 and OsCP1 can mutually regulate each other’s functions through their enzymatic activities during seed development.

## Discussion

In this study, we reported the roles of PDIL1-1 in the regulation of reproductive organ development in rice. In particular, we have tried to elucidate the mechanism of PDIL1-1 control on the amount and composition of various seed components through isolation of PDIL1-1 target and downstream proteins.

Two T-DNA insertion *PDIL1-1* mutants displayed the same phenotypic morphologies compared to the wild type, although T-DNA fragments were inserted at different loci within the *PDIL1-1* gene (Figure S2A and B). The mutant *PDIL1-1Δ* showed shrunken seeds and lower dry seed weight compared to the wild type, suggesting that it controls reproductive organ development. Expression analysis during seed development revealed that the transcript level of *PDIL1-1* gradually increased after flowering, reached a maximum at 10 DAF, and then sharply decreased to basal levels (Figure 5B), with detectable levels seen at mature seed phase (50 DAF) as previously shown [30], [31]. However, the PDIL1-1 protein level was similar during seed development (Figure 5C), suggesting that it is stabilized after translation. In eukaryotic cells, most proteins are post-translationally modified by various small and large molecules, and post-translational modifications are important mechanisms that modulate protein function and stability. PDIL1-1 can catalyze the post-translational formation, breakage, and isomerization of disulfide bonds, indicating that PDIL1-1 post-translationally controls the function and stability of its target proteins. Our 2-D gel analysis and mass spectrometry revealed at least five types of PDIL1-1 proteins in rice seed (Figure 5D). Therefore, these results strongly suggest that PDIL1-1 functions as an important regulator of reproductive organ development until maturation, and PDIL1-1 activity and stability can also be regulated by post-translational modifications.

The PDIL1-1 expression profiles during seed development also led us to investigate whether PDIL1-1 participates in the regulation of the composition and amount of carbohydrate, protein, and lipid in the seed, including the endosperm and aleurone layer. However, their contents in the *PDIL1-1Δ* mutant seeds are slightly increased or almost the same compared to those of the wild type (Table S3). Further investigation using electron microscopy clearly revealed that *PDIL1-1Δ* mutants have irregular, round-shaped starch granules (Figure S7), strongly indicating that the opaque phenotype of the *PDIL1-1Δ* mutant is caused by loose packing of round-shaped starch granules as recently reported [43], and the floury-white phenotype results from light scattering due to loose crystallization of round-shaped starch granules. However, starch granule crystalline properties, including the crystal type of starch and the chain length of amylopectin, were not significantly altered in the *PDIL1-1Δ* mutant seed at the whole endosperm level although they were somehow changed in the outer part of the endosperm (Figure 4, Figure S6). Previous results report that a change in carbohydrate metabolism resulted in an opaque endosperm of cereal crops [46]. Therefore, collectively, these data suggest that the opaque and floury-white endosperms of the *PDIL1-1Δ* mutant may be mediated by changes in carbohydrate metabolism due to a lack of PDIL1-1 activity. However, another possibility is that PDIL1-1 induces extensive post-translational changes, and the loss of this activity in *PDIL1-1Δ* leads to a floury-white phenotype during grain formation because the levels of many types of seed proteins are altered in the *PDIL1-1Δ* mutant (Table 2).

There are other possibilities that can explain the opaque endosperms of the *PDIL1-1Δ* mutant based on our western blot results and proteomic data. First, opaque endosperms can result from antioxidant enzymes. The highly expressed thioredoxins and peroxiredoxins can influence the expression of enzymes involved in carbohydrate and nitrogen metabolism. For example, thioredoxin regulates starch and protein breakdown as a scavenger of H_2_O_2_ in the starchy endosperm under oxidative stress [47]. ROS production causes opaque endosperms in cereal crops [46], and thioredoxins and peroxiredoxins participate in scavenging ROS induced from oxidative stress [48], [49]. In addition, sucrose synthase, phosphoglycerate kinase, 6-phosphogluconate dehydrogenase, alanine aminotransferase, and BiP are regarded as potential thioredoxin target proteins in starchy endosperms of rice as well as ER stress-induced proteins [50], [51]. In our proteomic data, these proteins are highly accumulated in *PDIL1-1Δ* mutant seeds, suggesting that the opaque endosperm of the *PDIL1-1Δ* mutant seeds may result from high levels of thioredoxin and peroxiredoxin due to PDIL1-1 loss. Additionally, ROS must be generated during maturation of the *PDIL1-1Δ* mutant seeds, and the *PDIL1-1* mutation stimulates ROS-scavenging mechanism through induction of expression or stabilization of these proteins. However, it remains unclear whether the opaque phenotype of the *PDIL1-1Δ* mutant is directly linked to the mechanism of ROS production and scavenging.

Second, the opaque endosperm could result from ER stress. Expression of genes related to ER stress and UPR signaling have been shown to be induced in the opaque endosperm of maize and rice [16], [51]. During grain filing in rice, abundant seed storage proteins are synthesized and stored within the ER. If improperly folded proteins are loaded beyond the capacity of quality control in the ER, then UPR signaling may be promoted, subsequently leading to ER stress and ROS production. Accumulation of irregularly folded storage proteins, including glutelin precursors, may impose a heavy burden on the ER, subsequently inducing ER stress and up/down-regulation of proteins, including BiP, as an ER stress sensor. There is evidence that changes in BiP protein levels induce ER stress [51], and BiP overexpression results in an opaque phenotype in the whole endosperm of rice [15]. In our study, the *PDIL1-1Δ* mutant seeds accumulated numerous glutelin precursors (Figure 2A). In addition, the BiP level was much higher in the *PDIL1-1Δ* mutant seeds [30], suggesting that the loss of PDIL1-1 may induce ER stress during grain filing, leading to an opaque endosperm.

Third, shrunken seeds with opaque endosperms can also result from an alteration in the composition of storage proteins and a distortion in the structure of protein bodies. Proteins are a main component of rice endosperms, although the levels are relatively low compared to carbohydrates, and protein is generally stored in protein bodies in the endosperm. Normally, prolamin is assembled on the ER-derived protein body (PB-ER) and remains as a stabilized PB-I form via intermolecular disulfide bonds. Glutelin precursor is synthesized within the cisternal ER (C-ER) and then exported via the Golgi apparatus to the protein storage vacuole (PSV), where it is proteolytically processed into acidic and basic subunits [52]. Rice PDI interacts with glutelin precursor in the C-ER and facilitates its maturation [31]. Thus, defective ER chaperone proteins, including PDI, may induce altered protein compositions in endosperms [15], [52]. In our study, the amount of seed proteins was much higher in the *PDIL1-1Δ* mutant seeds than in the wild type, and many types of seed proteins were highly accumulated in the *PDIL1-1Δ* mutant seeds compared to the wild type. Notably, *PDIL1-1Δ* mutant endosperms accumulate numerous irregular PB-I (Figure S3), which is conjugated with glutelin precursors and prolamin as shown in the PDIL1-1-lacking *esp2* mutant [30]. All of these results indicate that the *PDIL1-1* mutation causes an accumulation of seed proteins, suggesting a significant effect of PDIL1-1 on maturation and stability of seed proteins. However, whether abnormal protein bodies or the change of seed protein composition has direct effects on the endosperm phenotype of the *PDIL1-1Δ* mutant seeds remains unknown.

Han et al. very recently reported a similar result that T3612 mutant, which lacks PDIL1-1, promotes ER stress and alters starch metabolism, resulting in floury endosperms. However, this conclusion is based on changes on the transcript level of ER stress- and PCD-associated genes [43].

Fourth, the *PDIL1-1Δ* mutant phenotype can also be caused by the change of cysteine protease activity. Our study showed that PDIL1-1 strongly interacts with OsCP1. It has been recently reported that cysteine protease activity is inhibited by PDI in Arabidopsis [29]. Therefore, PDIL1-1 deficiency releases OsCP1 protease, which could result in digestion of enzymes related to carbohydrate metabolism as well as protein and lipid synthesis. However, we cannot rule out the possibility that PDIL1-1 deficiency may down-regulate OsCP1 protease activity, leading to the accumulation of various proteins and finally inducing the *PDIL1-1Δ* mutant phenotype.

Another important morphological feature of the *PDIL1-1Δ* mutant seed is that it has a much thicker aleurone layer than the wild type (Figure 3 A and B). It was reported that the cytoplasm of the mature aleurone cell is filled with organelles in barley. Most of these are PSVs that store amino acid building blocks to quickly produce enzymes to mobilize endosperms [53]. In addition, the change in storage protein composition has a large effect on total amino acid content in maize [54]. Various seed proteins are up-redulated in the *PDIL1-1Δ* mutant in our proteomic data (Table 2). Specifically, the levels of alanine amino transferase, tryptophan, and methionine synthases are elevated in *PDIL1-1Δ* mutant seeds (Table 2), which may contribute to the deposition of more seed reserves in the aleurone layer of the *PDIL1-1Δ* mutant. From this result, we carefully infer that the thick aleurone layer of the *PDIL1-1Δ* mutant seeds could be caused by increased PSV through elevated levels of enzymes that provide amino acid building blocks more quickly.

There are many types of PDIL proteins in rice [27], which may have distinct redox activities. For example, PDIL1-1 refolded, reduced, and denatured RNase and α-globulin with a higher efficiency than did PDIL2-3 in a GSH/GSSG ratio-dependent manner, but PDIL2-3 exhibited a significantly higher sulfhydryl oxidase activity to form nonnative intermolecular disulfide bonds when reduced, denatured α-globulin mutant was used as substrate [32]. In addition, PDIL2-3 proteins have been suggested to interact with BiP protein because BiP interacts with P5, and P5 and PDIL2-3 have high sequence similarity [32], suggesting that elevated levels of BiP in the *PDIL1-1Δ* mutant can regulate PDIL2-3 activity. These results indicate that the opaque phenotype can be caused by levels and functions of other PDIL proteins through a regulatory effect of PDIL1-1.

In conclusion, loss of the PDIL1-1 protein causes changes in the amount and composition of proteins involved in synthesis of various components including free sugars in rice seed. Consequently, the *PDIL1-1Δ* mutant has shrunken seeds with an opaque endosperm and a thick aleurone layer. Further analysis of its upstream and target proteins and characterization of their mutants along with the *PDIL1-1Δ* mutant will provide clues to understanding the various roles of PDIL1-1 in rice development.

## Supporting Information

Figure S1 Isolation of another *PDIL1-1* mutant allele. (A) Identification of the T-DNA insertion site in PGF_2B-80111.R mutant allele by PCR. Independent transgenic lines were analyzed by PCR using two sets of primers, as shown in Table S1. In non-transgenic lines, 1,031-bp fragments were amplified by PCR with LP and RP, whereas approximately 700-bp fragments were amplified by BP and RP in transgenic homozygote lines. (B) Identification of 2B-80111 mutant allele by western blot. Total seed proteins extracted from the lines described in (A) were separated by SDS-PAGE and examined by western blot with an anti-PDIL1-1 antibody.
(TIF)

Figure S2 (A) Schematic diagram of two T-DNA insertion mutant alleles. T-DNA was inserted in the tenth exon for PFG_1B-16041.R mutant allele and in the first intron for PFG_2B-80111.R mutant allele, respectively. (B) T-DNA insertion sites are indicated by arrowheads in the nucleotide sequences. Blue and black letters indicate introns and exons, respectively.
(TIF)

Figure S3 Protein bodies of *PDIL1-1Δ* mutant endosperm. Mature seeds of the WT and *PDIL1-1Δ* mutant were harvested, hand-sectioned with a razor blade, and then analyzed by TEM. *PDIL1-1Δ* mutants show irregular, prolamin and glutelin-containing PBI (PB-I). Bar, 0.5 µm.
(TIF)

Figure S4 Phenotype of *PDIL1-1Δ* mutant seeds. (A) Seed morphology was observed on an illuminator. Mature seeds of the WT and *PDIL1-1Δ* mutant were hand-sectioned with a razor blade: (a) whole seeds; (b) vertically sectioned seeds; (c) transversely sectioned seeds. (B) Seed morphology was observed by light microscopy. Mature seeds of the WT and *PDIL1-1Δ* mutant were hand-sectioned with a razor blade: (a) traverse sections; (b) vertical sections. *PDIL1-1Δ* mutants show floury-white endosperms. Bar, 0.3 cm.
(TIF)

Figure S5 Iodine staining analysis of *PDIL1-1Δ* mutant seeds. Mature seeds of the WT and *PDIL1-1Δ* mutant were cut for transverse-view. Starch granules were stained with iodine solution.
(TIF)

Figure S6 X-ray diffraction analysis of the *PDIL1-1Δ* mutant seeds. Polished mature seeds were powdered and analyzed by X-ray diffractometry. The two-theta angle (2θ) ranging from 4.0° to 40.0° was scanned to obtain values that overlapped for comparison of the two samples. Gray indicates WT and black indicates the *PDIL1-1Δ* mutant.
(TIF)

Figure S7 Starch granules of *PDIL1-1Δ* mutant endosperm. Mature seeds of the WT and *PDIL1-1Δ* mutant were harvested, hand-sectioned with a razor blade, and then analyzed by SEM. *PDIL1-1Δ* mutants show round-edged starch granules. Bar, 20 µm.
(TIF)

Table S1 List of primers used for selection of *PDIL1-1* mutants.
(DOCX)

Table S2 List of primers used for real time RT-PCR and northern blot.
(DOCX)

Table S3 Comparison of starch, amylose, and lipid amounts between the WT and *PDIL1-1Δ* mutant.
(DOCX)

Table S4 Protein identification by MALDI-TOF mass spectrometry.
(DOCX)
