# Supplementary figures and images for: Protein Disulfide Isomerase-Like Protein 1-1 Controls Endosperm Development through Regulation of the Amount and Composition of Seed Proteins in Rice
Source: PLoS One. 2012 Sep 6;7(9):e44493. doi: 10.1371/journal.pone.0044493 (PMC3435311; doi:10.1371/journal.pone.0044493)

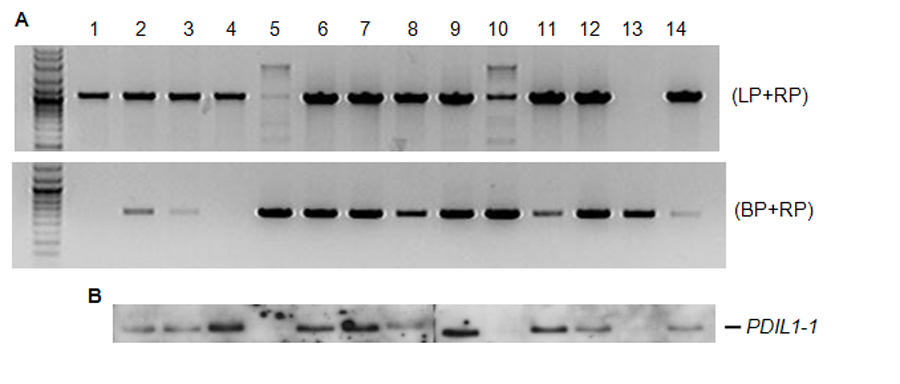

Supplement: Figure S1 — Isolation of another PDIL1-1 mutant allele. (A) Identification of the T-DNA insertion site in PGF_2B-80111.R mutant allele by PCR. Independent transgenic lines were analyzed by PCR using two sets of primers, as shown in Table S1. In non-transgenic lines, 1,031-bp fragments were amplified by PCR with LP and RP, whereas approximately 700-bp fragments were amplified by BP and RP in transgenic homozygote lines. (B) Identification of 2B-80111 mutant allele by western blot. Total seed proteins extracted from the lines described in (A) were separated by SDS-PAGE and examined by western blot with an anti-PDIL1-1 antibody. (TIF) [file pone.0044493.s001.tif]

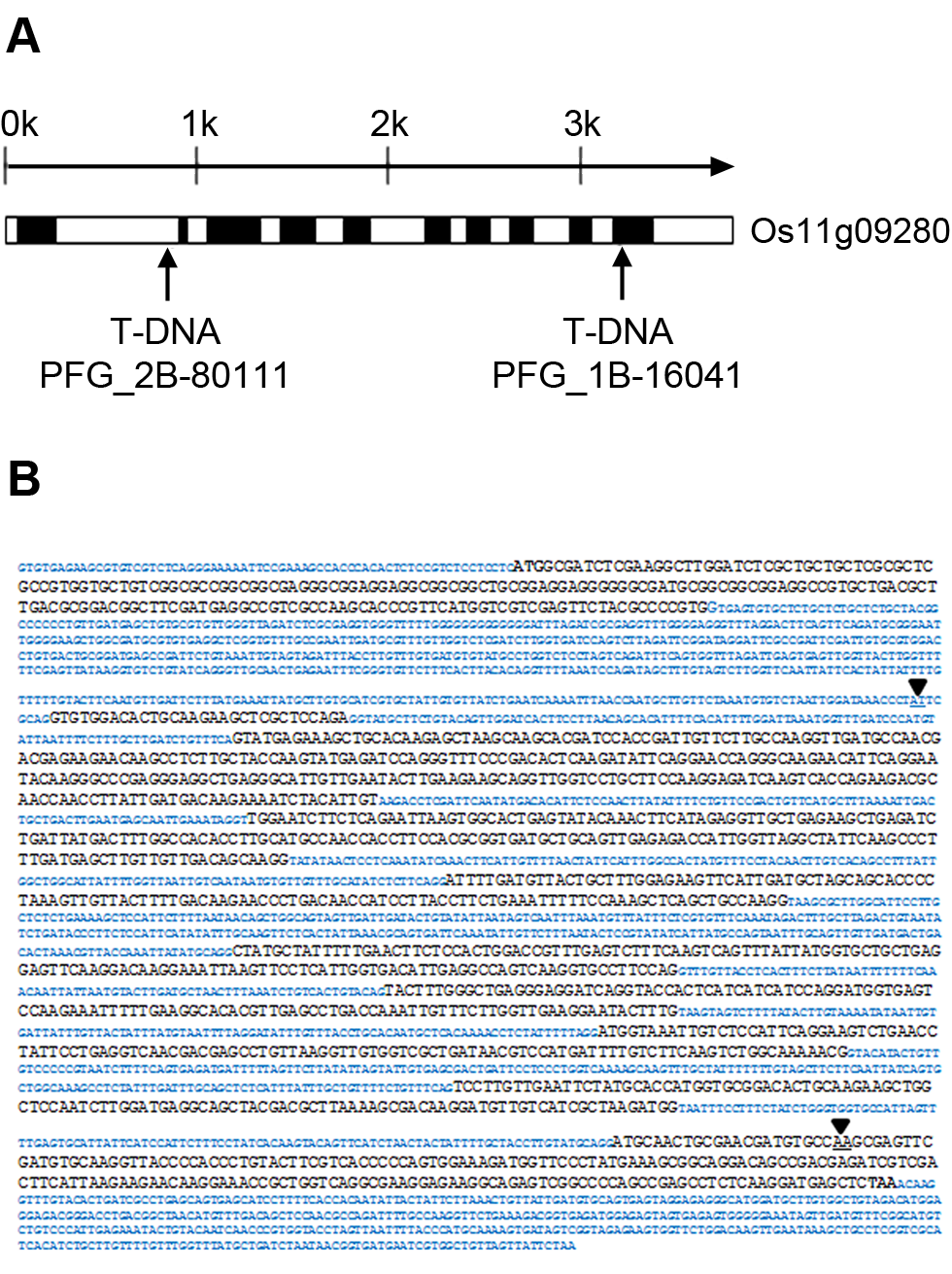

Supplement: Figure S2 — (A) Schematic diagram of two T-DNA insertion mutant alleles. T-DNA was inserted in the tenth exon for PFG_1B-16041.R mutant allele and in the first intron for PFG_2B-80111.R mutant allele, respectively. (B) T-DNA insertion sites are indicated by arrowheads in the nucleotide sequences. Blue and black letters indicate introns and exons, respectively. (TIF) [file pone.0044493.s002.tif]

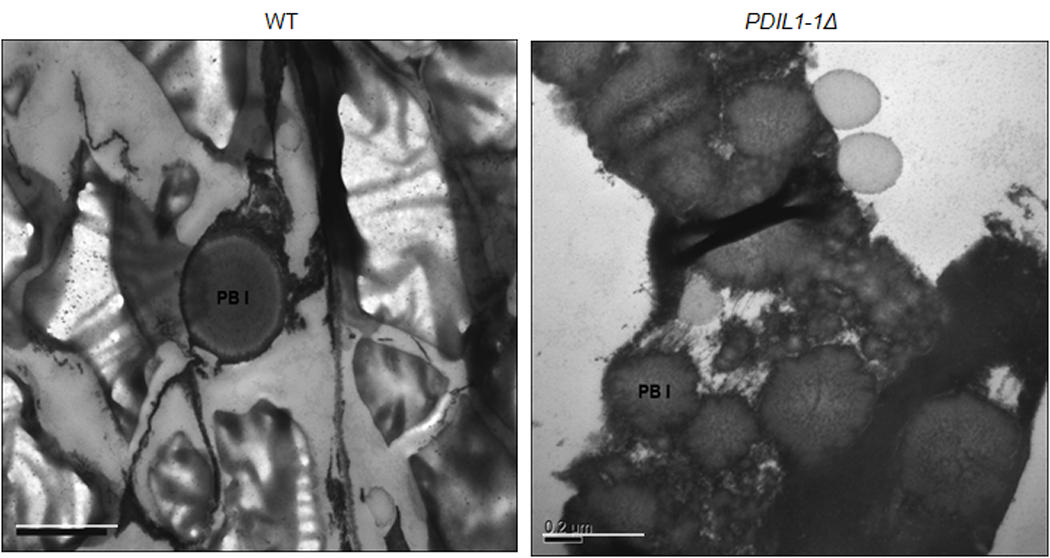

Supplement: Figure S3 — Protein bodies of PDIL1-1Δ mutant endosperm. Mature seeds of the WT and PDIL1-1Δ mutant were harvested, hand-sectioned with a razor blade, and then analyzed by TEM. PDIL1-1Δ mutants show irregular, prolamin and glutelin-containing PBI (PB-I). Bar, 0.5 µm. (TIF) [file pone.0044493.s003.tif]

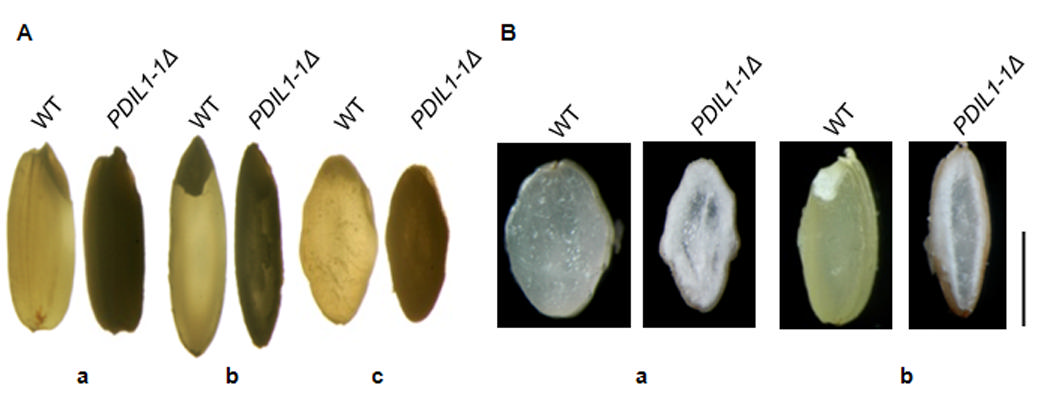

Supplement: Figure S4 — Phenotype of PDIL1-1Δ mutant seeds. (A) Seed morphology was observed on an illuminator. Mature seeds of the WT and PDIL1-1Δ mutant were hand-sectioned with a razor blade: (a) whole seeds; (b) vertically sectioned seeds; (c) transversely sectioned seeds. (B) Seed morphology was observed by light microscopy. Mature seeds of the WT and PDIL1-1Δ mutant were hand-sectioned with a razor blade: (a) traverse sections; (b) vertical sections. PDIL1-1Δ mutants show floury-white endosperms. Bar, 0.3 cm. (TIF) [file pone.0044493.s004.tif]

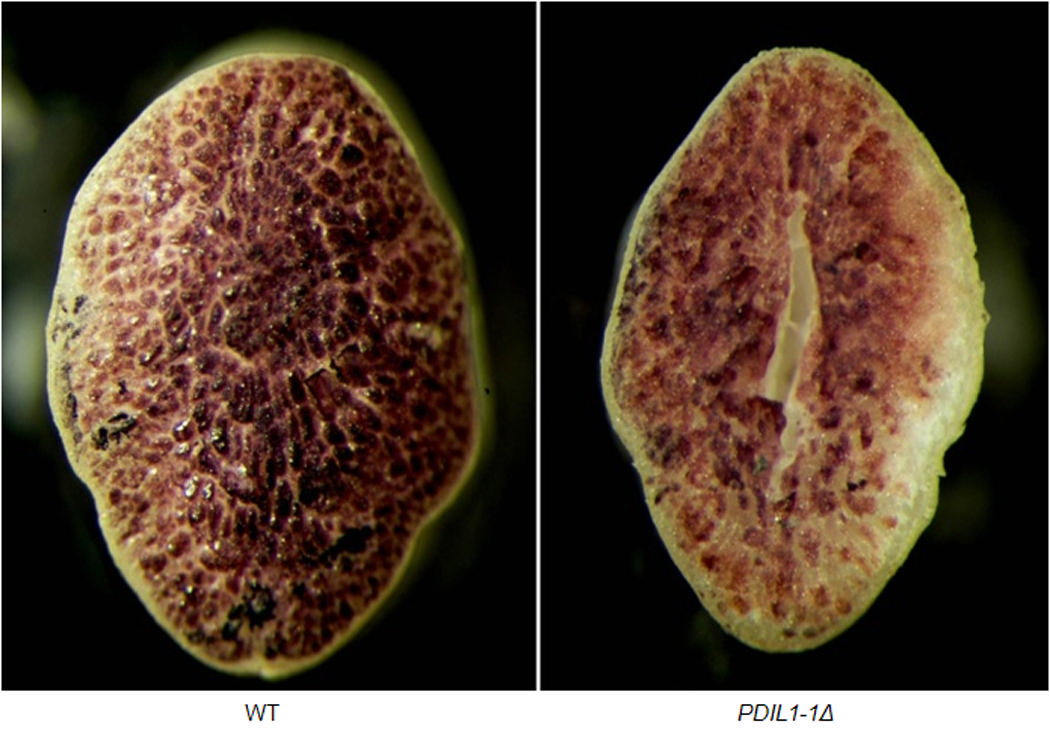

Supplement: Figure S5 — Iodine staining analysis of PDIL1-1Δ mutant seeds. Mature seeds of the WT and PDIL1-1Δ mutant were cut for transverse-view. Starch granules were stained with iodine solution. (TIF) [file pone.0044493.s005.tif]

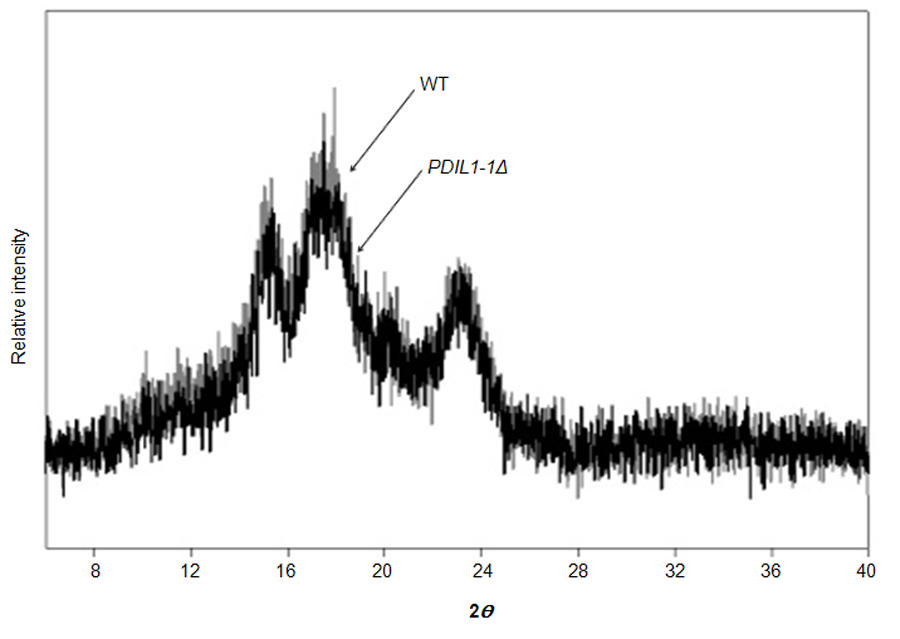

Supplement: Figure S6 — X-ray diffraction analysis of the PDIL1-1Δ mutant seeds. Polished mature seeds were powdered and analyzed by X-ray diffractometry. The two-theta angle (2θ) ranging from 4.0° to 40.0° was scanned to obtain values that overlapped for comparison of the two samples. Gray indicates WT and black indicates the PDIL1-1Δ mutant. (TIF) [file pone.0044493.s006.tif]

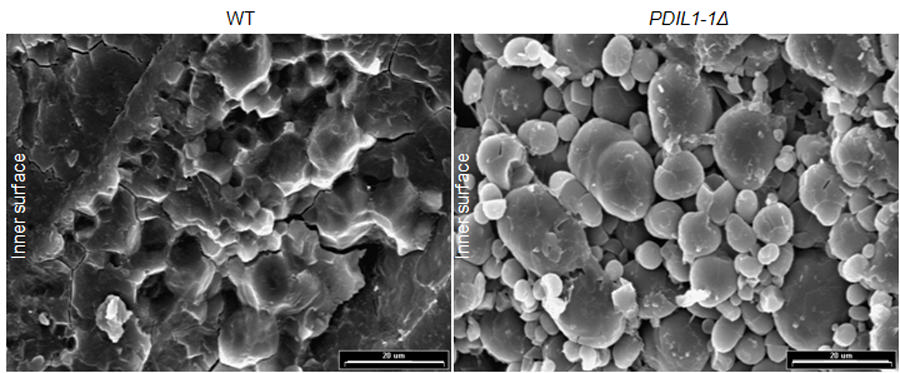

Supplement: Figure S7 — Starch granules of PDIL1-1Δ mutant endosperm. Mature seeds of the WT and PDIL1-1Δ mutant were harvested, hand-sectioned with a razor blade, and then analyzed by SEM. PDIL1-1Δ mutants show round-edged starch granules. Bar, 20 µm. (TIF) [file pone.0044493.s007.tif]
