# Supplementary material for: Protein Disulfide Isomerase-Like Protein 1-1 Controls Endosperm Development through Regulation of the Amount and Composition of Seed Proteins in Rice
Source: PLoS One. 2012 Sep 6;7(9):e44493. doi: 10.1371/journal.pone.0044493 (PMC3435311; doi:10.1371/journal.pone.0044493)
Supplement: Table S1 — List of primers used for selection of PDIL1-1 mutants. (DOCX) [file pone.0044493.s008.docx]

**Table S1. List of primers used for selection of *PDIL1-1* mutants**.

| Primer sequences for selection of PFG_1B-16041 and PFG_2B-80111 mutants (PCR Product: 1,229bp (LP/RP), 607-907 bp (BP/RP) for PFG_1B-16041, 1031bp (LP/RP), 508-808 bp (BP/RP) for PFG_2B-80111) | | |
| --- | --- | --- |
| PFG_1B-16041 | Left primer (LP) | 5’-TGCAAAGACGTGGAGTTCAG-3’ |
|  | Right primer (RP) | 5’-TGTTAAGGTTGTGGTCGCTG-3’ |
|  | Border primer (BP) | 5’-CCACAGTTTTCGCGATCCAGACTG-3’ |
| PFG_2B-80111 | Left primer (LP) | 5’-GTGAGTGTGCTCTGCTCTGC-3’ |
|  | Right primer (RP) | 5’-TTGCGTCTTCTGGTGACTTG-3’ |
|  | Border primer (BP) | 5’-CCACAGTTTTCGCGATCCAGACTG-3’ |
