# Supplementary material for: Protein Disulfide Isomerase-Like Protein 1-1 Controls Endosperm Development through Regulation of the Amount and Composition of Seed Proteins in Rice
Source: PLoS One. 2012 Sep 6;7(9):e44493. doi: 10.1371/journal.pone.0044493 (PMC3435311; doi:10.1371/journal.pone.0044493)
Supplement: Table S2 — List of primers used for real time RT-PCR and northern blot. (DOCX) [file pone.0044493.s009.docx]

**Table S2. List of primers used for real time RT-PCR and northern blot**.

| Primer sequences for real time PCR and northern blot (PCR product: 201 bp for PDIL1-1, 257 bp for OsCP1, 157 bp for Os05g0108600, 203 bp for Os01g0971400, 456 bp for PDIL1-1 northern blot) | | | |
| --- | --- | --- | --- |
| RT-PCR | PDIL1-1 | Forward primer (F) | 5’-AACGATGTGCCAAGCGAGTTCGAT-3’ |
|  |  | Reverse primer (R) | 5’-TTAGAGCTCATCCTTGAGAGGCTC-3’ |
| RT-PCR | OsCP1 | Forward primer (F) | 5’-CCTGCTGCTGCAACTACG-3’ |
|  |  | Reverse primer (R) | 5’-CTATACTAGATCTTCCCT-3’ |
| RT-PCR | Os05g0108600 | Forward primer (F) | 5’-AACCACATCGACGACATCAA-3’ |
|  |  | Reverse primer (R) | 5’-TCCTCCGACGAGTAGTGCTT-3’ |
| RT-PCR | Os01g0971400 | Forward primer (F) | 5’-AGGAGTCCTACCCGTACCTGAT-3’ |
|  |  | Reverse primer (R) | 5’-GAACTGGAAGTTCCTGCCTGAG-3’ |
| Northern blot | PDIL1-1 | Forward primer (F) | 5’-TCTACGAATTCATGACTGCTTTGGAGAAGTTC-3’ |
|  |  | Reverse primer (R) | 5’-TCTACGAGCTCTTAAGCGACCACAACCTTAACAGG-3’ |
