# Supplementary material for: Protein Disulfide Isomerase-Like Protein 1-1 Controls Endosperm Development through Regulation of the Amount and Composition of Seed Proteins in Rice
Source: PLoS One. 2012 Sep 6;7(9):e44493. doi: 10.1371/journal.pone.0044493 (PMC3435311; doi:10.1371/journal.pone.0044493)
Supplement: Table S3 — Comparison of starch, amylose, and lipid amounts between the WT and PDIL1-1Δ mutant. (DOCX) [file pone.0044493.s010.docx]

**Table S3. Comparison of starch, amylose, and lipid amounts between the WT and *PDIL1-1Δ* mutant**.

|  | Starch (% milled rice weight) | Amylose (% milled rice weight) | Lipid (% milled rice weight) |
| --- | --- | --- | --- |
| WT | 86.9±0.1 | 18.1±0.4 | 2.4±0.09 |
| *PDIL1-1Δ* | 88.4±0.7 | 19.1±0.2 | 2.5±0.13 |
| p-value  (two tail, t-test) | 0.68 | 0.43 | 0.62 |

The numerical values represent the mean of three independent experiments, and values are expressed as means ± standard deviation.
