# Supplementary material for: Protein Disulfide Isomerase-Like Protein 1-1 Controls Endosperm Development through Regulation of the Amount and Composition of Seed Proteins in Rice
Source: PLoS One. 2012 Sep 6;7(9):e44493. doi: 10.1371/journal.pone.0044493 (PMC3435311; doi:10.1371/journal.pone.0044493)
Supplement: Table S4 — Protein identification by MALDI-TOF mass spectrometry. (DOCX) [file pone.0044493.s011.docx]

**Table S4. Protein identification by MALDI-TOF mass spectrometry**.

| Spot no. | Matched Protein | Accession no. | Experimental size (kDa/pI) | Calculated size (kDa/pI) | Z-score | Coverage (%) | Peptides identified by MALDI-TOF mass spectrometry |
| --- | --- | --- | --- | --- | --- | --- | --- |
| 710 | Protein disulfide isomerase | AY987391 | 58.99/4.38 | 56.97/5.24 | 1.33 | 35 | SEPIPEVNDEPVKVVVADNVHDFVFK |
| 711 | Protein disulfide isomerase | AY987391 | 60.13/4.43 | 56.97/5.24 | 2.33 | 38 | LAPILDEAATTLKSDEDVVIAK |
| 602 | Protein disulfide isomerase | AY987391 | 60.88/4.41 | 56.97/5.24 | 2.19 | 38 | SEPIPEVNDEPVKVVVADNVHDFVFK |
| 712 | Protein disulfide isomerase | AY987391 | 62.18/4.46 | 56.97/5.24 | 2.03 | 43 | MDATANDVPSEFDVQGYPTLYFVTPSGK |
| 714 | Protein disulfide isomerase | AY987391 | 61.61/4.68 | 56.97/5.24 | 2.30 | 48 | MDATANDVPSEFDVQGYPTLYFVTPSGK |
